# Animal Vaccination

**Published:** 1882-01-20

**Authors:** William B. Carpenter

**Affiliations:** London, England


### ANIMAL VACCINATION.

BY WILLIAM B. CARPENTER, M. D., OF LONDON, ENGLAND.

It becomes possible to affect sheep and cattle with a form of anthrax disease so mild as to bear much the same relation to the severer forms that cowpox bears to smallpox; and for this artificial affection with the mitigated disorder, Pasteur uses the term "vaccination." The question that now arises—to which the whole previous investigation has led up—is the most important of all: Does this "vaccination" with the mild virus afford the same protection against the action of the severe, that is imparted by cowpox vaccination against smallpox? To this question affirmative answers were last year obtained by Professor Greenfield (on Professor Burdon-Sanderson's suggestion) in regard to bovine animals, and by M. Toussaint in regard to sheep and dogs; the former, when "vaccinated" from rodents, and the latter from fluids "cultivated" outside the living body after a method devised by M. Toussaint, proving themselves incapable of being infected with any form of anthrax disease, though repeatedly inoculated with the malignant virus, and remaining free from all disorder, either constitutional or local.

The same result having been obtained from experiments made by Pasteur himself, probably about the same date, with charbon virus cultivated in the manner previously described, it was deemed expedient by one of the Provincial Agricultural Societies of France that this important discovery should be publicly demonstrated on a great scale. Accordingly, a farm and a flock of fifty sheep having been placed at M. Pasteur's disposal, he "vaccinated" twenty-five of the flock (distinguished by a perforation of their ears), with the mild virus on the 3d of May last, and repeated the operation on the 17th of the same month. The animals all passed through a slight indisposition, but at the end of the month none of them were found to have lost either fat, appetite, or liveliness. On the 31st of that month, all the fifty sheep, without distinction, were inoculated with the strongest charbon virus, and M. Pasteur predicted that on the following day the twenty-five sheep inoculated for the first time would all be dead, while those protected by previous "vaccination" with the mild virus would be perfectly free from even slight indisposition. A large assemblage of agricultural authorities, cavalry officers, and veterinary surgeons having met at the field the next afternoon (June 1st), the result was found to be exactly in accordance with M. Pasteur's predictions. At two o'clock twenty-three of the "unprotected" sheep were dead; the twenty-fourth died within an hour, and the twenty-fifth an hour afterward. But the twenty-five "vaccinatəd" sheep were all in perfectly good condition; one of them, which had been designedly inoculated with an extra dose of the poison, having been slightly indisposed for a few hours, but having then recovered. The twenty-five carcasses were then buried in a selected spot, with a view to the further experimental testing of the poisonous effect produced upon the grass which will grow over their graves. But the result, says the reporter of the *Times* (June 2d), "is already certain; and the agricultural public now know that an infallible preventive exists against the charbon poison, which is neither costly nor difficult, as a single man can inoculate a thousand sheep in a day." I have since learned that this protection is being eagerly sought by the French owners of flocks and herds; and, if any severe epidemic of the same kind were to break out in this country, our own agriculturists would probably show themselves quite ready to avail themselves of it.—[Popular Science monthly for December.

